# Microbial indoor air pollution in Delhi Metropolitan City is attributable to severe respiratory and general health effects among residents

**DOI:** 10.3389/fpubh.2025.1626827

**Published:** 2025-07-24

**Authors:** Pradeep Kumar, Rajeev Singh

**Affiliations:** ^1^Department of Environmental Studies, Satyawati College, University of Delhi, New Delhi, India; ^2^Department of Agricultural and Biosystems Engineering, South Dakota State University, Brookings, SD, United States; ^3^Department of Environmental Science, Jamia Millia Islamia, New Delhi, India

**Keywords:** indoor air quality, questionnaire survey, respiratory problems, bacteria, fungi

## Abstract

Indoor air quality plays a crucial role in the health and well-being of residents. Delhi, known as one of the most polluted cities globally, often receives insufficient attention in managing and mitigating related health impacts. This study isolated, characterized, and assessed microbial indoor air quality (bioaerosols) using multiproxy approaches and correlated findings with associated health effects. The spatial variation of bacterial aerosols showed irregular patterns, increasing from winter to summer and decreasing in fall; fungal aerosols consistently increased from winter to fall. Bacterial aerosol concentrations ranged from 730 to 5,300 CFU/m^3^, while fungal concentrations were between 1,330 and 6,050 CFU/m^3^, significantly exceeding the recommended limits. The size distribution of fungal aerosols varied across seasons, with higher concentrations in the 4th and 5th stages of the sampler. Several airborne bacterial and fungal genera, including *Staphylococcus*, *Streptococcus*, *Micrococcus*, *Aspergillus*, *Penicillium*, and *Cladosporium*, were identified in homes. Health effects were most pronounced in winter followed by fall, with symptoms such as headaches, eye irritation, allergic rhinitis, coughing, and sneezing being common. As per this study, there may be a correlation between indoor bioaerosol concentrations, seasonal variations, and health outcomes, though further in-depth *in vitro*, exposure assessment, and epidemiological studies are necessary to substantiate these findings.

## Graphical abstract

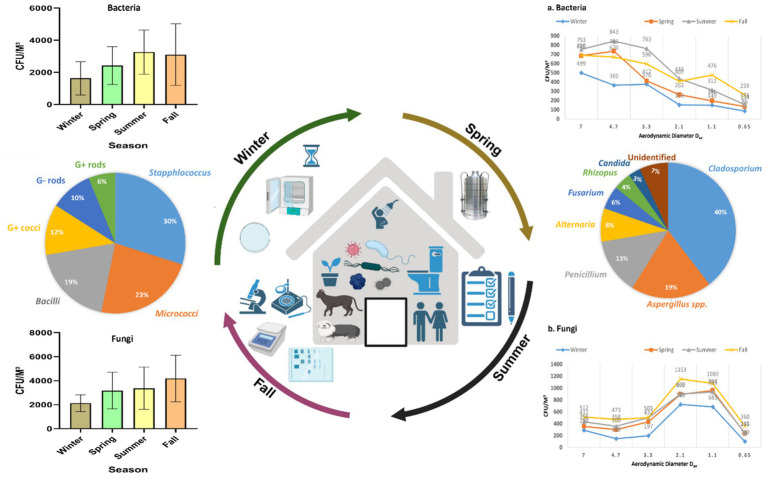

## 1 Introduction

Bioaerosols are airborne particles of biological origin, including both pathogenic and non-pathogenic bacteria, fungal spores, viruses, and droplets released during human activities such as coughing and sneezing. With increasing awareness of the potential dangers posed by biological agents, there is growing concern regarding bioaerosol exposure, particularly in confined environments. This exposure can significantly impact both individual and public health, contributing to conditions such as allergies, asthma, respiratory infections, and even cancer (1–6, 59).

Numerous research studies have indicated that bioaerosol concentrations play a significant role in the onset of indoor allergies, asthma, bronchitis, laryngitis, and other respiratory health issues (3, 7–9, 59). Among airborne microorganisms, fungi such as *Aspergillus, Curvularia*, and *Fusarium* are more likely to trigger respiratory allergies and asthma than bacteria (10). Additionally, respirable bioaerosol particles smaller than 2.5 microns can penetrate deep into the alveoli, leading to potential long-term health consequences (11).

Given that people spend nearly 90% of their time indoors, poor indoor air quality can have an even greater impact on health compared to outdoor air pollution (8). Communities near industrial sites may be at greater risk due to elevated levels of pollutants, including bioaerosols, which contribute to various health problems. Variations in indoor air pollutants such as formaldehyde, total volatile organic compounds (TVOCs), and particulate matter (PM) have been associated with respiratory, digestive, skin, eye, and ear-related health issues, particularly among workers and students in poorly ventilated environments (12, 59). Overcrowded residential areas with inadequate ventilation further exacerbate these health risks.

Bioaerosol concentrations can range from a few colony-forming units (CFU) per cubic meter in typical residential environments to billions (10^9^ CFU/m^3^) near waste disposal sites. These concentrations fluctuate with seasonal changes, which in turn influence bioaerosol abundance, diversity, viability, and community composition. Meteorological factors such as temperature, humidity, and wind speed also play a critical role in bioaerosol dispersion and persistence (58).

To mitigate the risks associated with indoor bioaerosol exposure, several national and international organizations have established guidelines or recommendations for acceptable bioaerosol concentrations (13, 14). For instance, the World Health Organization (WHO) has recommended that fungal bioaerosol levels in residential areas should not exceed 500 CFU/m^3^ (14). The China Centers for Disease Control and Prevention set a limit of 2,500 CFU/m^3^ for bioaerosol concentrations in homes, while the American Conference of Governmental Industrial Hygienists (ACGIH) established a safe threshold of 1,000 CFU/m^3^ for fungal bioaerosols (15, 53). In South Korea, the Ministry of Environment mandates limits of 800 CFU/m^3^ for bacterial aerosols and 500 CFU/m^3^ for fungal aerosols in healthcare and daycare facilities where immunocompromised individuals are present (7, 16). However, existing regulations focus solely on bioaerosol concentration without accounting for their size, which significantly influences health risks. Bioaerosols can range in size from a few nanometers to over 100 micrometers, with PM2.5 and PM10 being particularly relevant categories for respiratory exposure (17).

Airborne microorganisms are small enough to bypass inertial filtration in the upper airways and can reach the alveolar region of the lungs, leading to potential health hazards (18–20). While bioaerosol standards and concentration guidelines provide a framework for assessing contamination levels, evaluating their actual health risks remains a challenge (7, 21).

In Delhi, India, a unique study is being conducted to investigate the health effects of biological and non-biological indoor air pollutants. This study aims to assess the seasonal variation in bioaerosols in residential houses with poor ventilation and facilities, correlating microbial concentration with observed health effects. The findings from this study will provide valuable insights into the impact of indoor air quality on human health, highlighting the need for improved regulatory measures and mitigation strategies.

## 2 Materials and methods

### 2.1 Sites location and description

This study was carried out in Delhi, the capital of India, which ranks among the most densely populated cities globally. Situated between the states of Uttar Pradesh and Haryana, Delhi is divided by the Yamuna River and features the Aravali hill ranges. The city experiences a warm, semi-arid climate with subtropical humidity and dry winters. According to IQAIR’s 2024 report, Delhi was the third most polluted city in the world in 2023 (57). This study was conducted throughout all four seasons- winter, spring, summer, and fall to assess the impact of poor air quality on human health. The research focused on densely populated areas, specifically the slum regions near Ashok Vihar and Azadpur in North Delhi. The selected houses for sampling were predominantly overcrowded, poorly ventilated, and lacked proper hygiene. The houses surveyed exhibited substantial variation in size, with room dimensions ranging from less than 100 square feet to significantly larger spaces. The average household occupancy was approximately six people. A total of 336 houses were sampled, with 84 houses assessed in each season. The typical floor plan consisted of one to two multifunctional rooms that served as both bedroom and living space, accompanied by a kitchen and a bathroom. Most dwellings were one to two stories in height. Natural ventilation through windows was the primary mode of air exchange in the majority of homes. Mechanical ventilation systems were largely absent during the field survey and air sampling period. Although direct measurements of air changes per hour (ACH) were not conducted, observational evidence indicated considerable limitations in natural ventilation, such as obstructed or sealed windows and inadequate cross-ventilation, which likely contributed to compromised indoor air quality. Visible and olfactory signs of dampness and mold were frequently noted across numerous dwellings. Furthermore, most houses did not contain indoor pets or plants, reducing additional sources of biological variation in indoor air quality.

### 2.2 Biological sampling details

**Figure 1:**
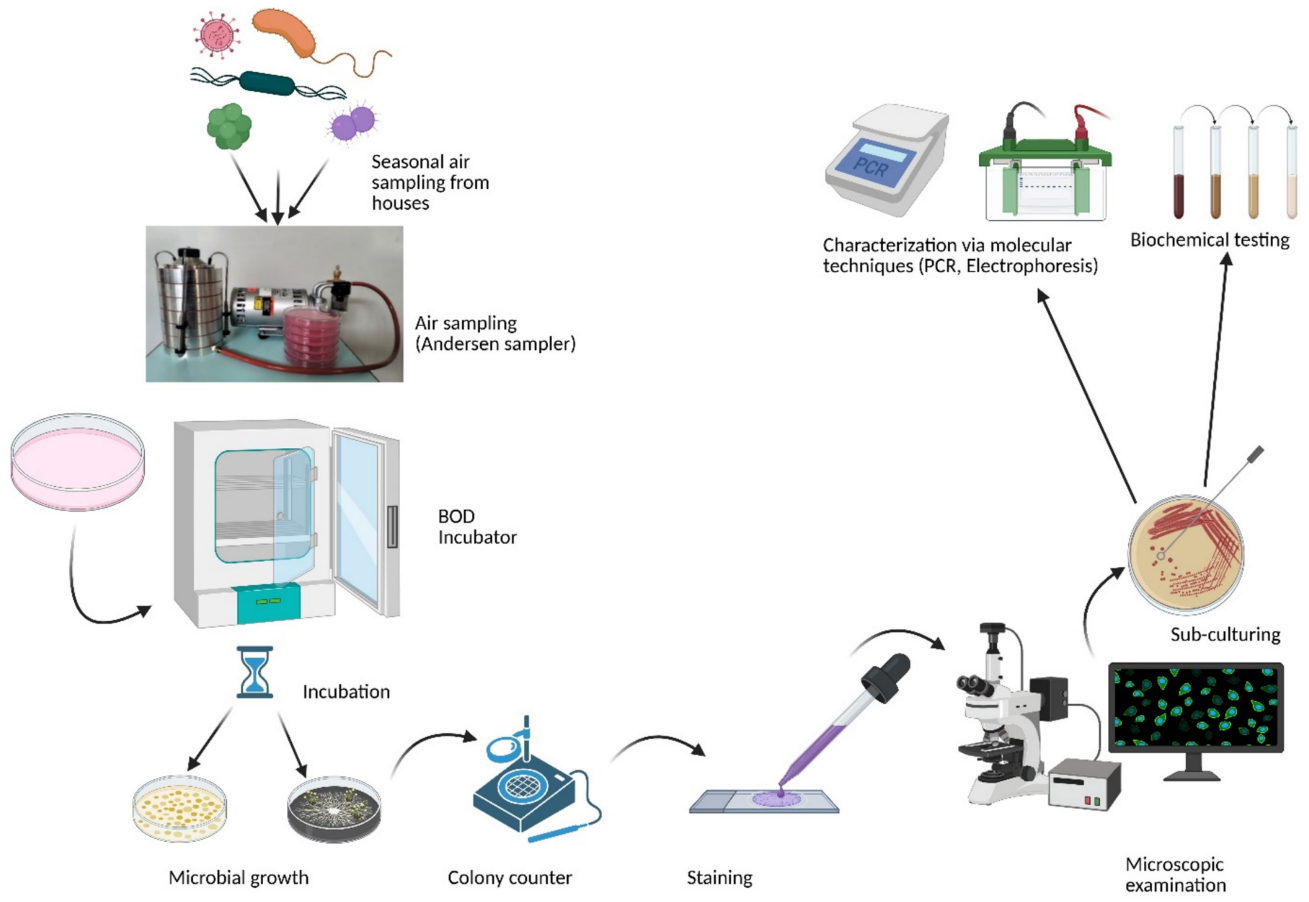
Simplified overview of the methodology used for bioaerosol sampling and analysis (Created with BioRender.com).

For bacterial aerosols, Tryptic Soy Agar (HiMedia, India) supplemented with Cycloheximide (an antifungal agent) was employed, while for fungal aerosols, Sabouraud Dextrose Agar (SDA) (HiMedia, India) supplemented with Rose Bengal Dye (an antibacterial agent) was used for sampling and culturing. Prior to sampling, various culture media were evaluated for their effectiveness in collecting indoor bioaerosols, and the selected media proved most suitable for supporting the growth of a wide range of indoor culturable bioaerosols. Biological air sampling was conducted using an Andersen Six-Stage Impactor (Tisch Environmental, USA) operating at an airflow rate of 28.3 L/min. For each sampling session, six sterile 90 mm petri plates containing the specified culture media were prepared. Sampling durations were calibrated and determined in advance based on the characteristics of each sampling site. Furthermore, the sampler was used at an inhaling level of 1.5 m. After aseptic exposure, the plates were transferred to the laboratory, incubated, and the colony growth was quantified using a colony counter to determine the colony-forming units (CFU). Bacterial petri plates were incubated at 37°C for 1 to 3 days, whereas fungal plates were incubated at 28°C for 1 to 2 weeks. Growing colonies were measured and reported using colony-forming units (CFUs) per cubic meter of air. Overview of the bioaerosol sampling methodology is depicted in (Figure 1). The following formula was used to determine CFU/m^3^ in current study (3).


(1) $$\begin{array}{l} \left(\text{Number of colonies}\times 1,000\right)\\ /(\text{Sampling time}\;(\min)\times \text{Velocity of airflow}) \end{array}$$

**Figure 2:**
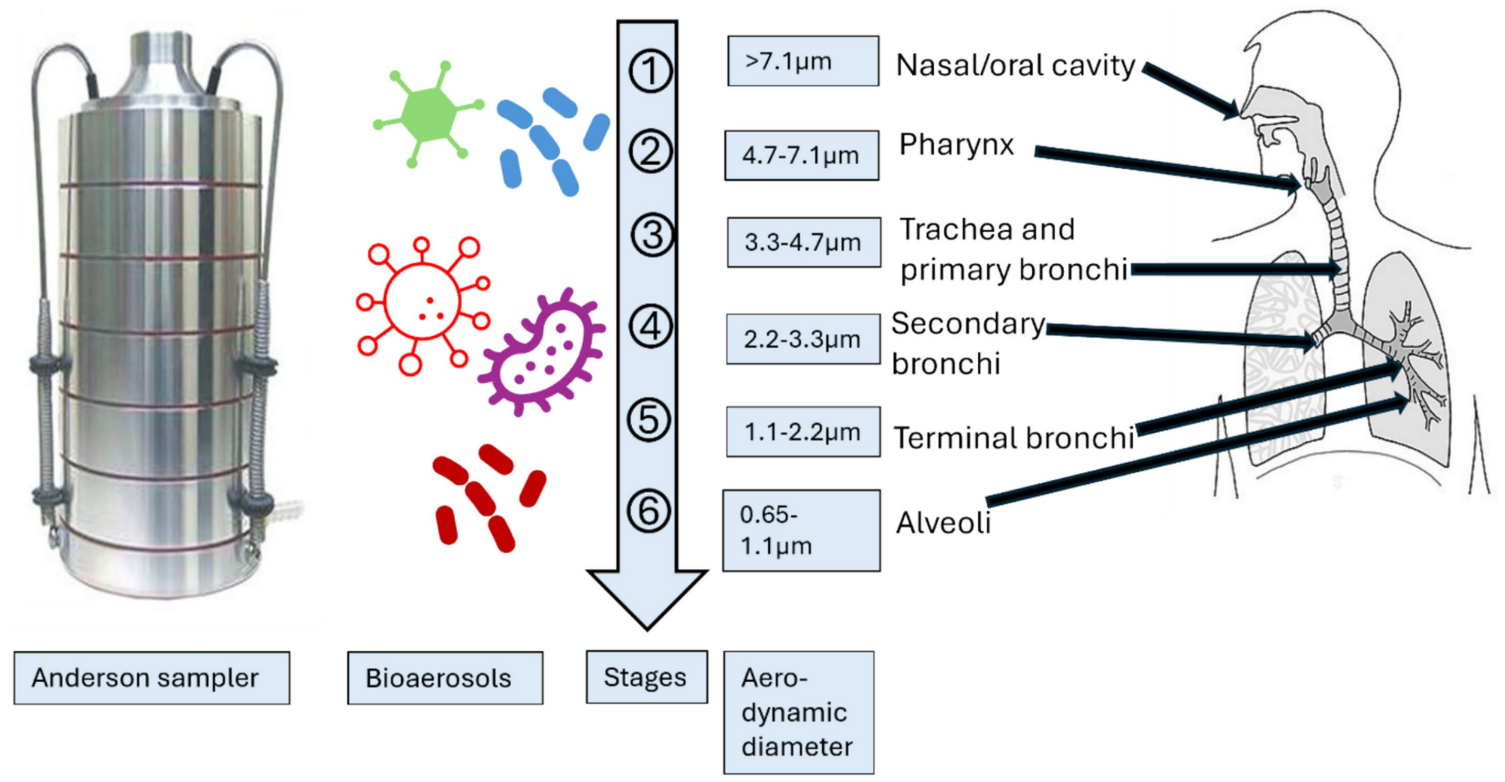
Schematic presentation of the comparison of size distribution of Andersen impactor with human respiratory system (54). (Created with BioRender.com).

The purpose of isolating and characterizing bioaerosols of different sizes was to investigate how airborne particles of varying sizes can infiltrate different segments of the human respiratory system (Figure 2). Andersen proposed that particles with a size of 7 μm or larger (1st stage) would adhere to the pre-separator, particles ranging from 4.7 to 7.0 μm (2nd stage) would be retained in the pharynx, particles measuring 3.3–4.7 μm (3rd stage) would settle in the trachea and primary bronchi, particles within the range of 3.3–2.1 μm (4th stage) would reach the secondary bronchi, particles of 2.1–1.1 μm (5th stage) would enter the terminal bronchi, and particles measuring 1.1–0.65 μm (6th stage) would ultimately reach the alveoli of the lungs (3, 22, 54, 59).

### 2.3 Identification and quantification of bacterial and fungal aerosols

Following the successful collection and culture of bacterial and fungal aerosols, methods for identification were carried out. For instance, of bacteria, isolated samples were identified using gram staining, cell morphology, colony morphology, and other macrobiological and microbiological characteristics. These traits were then compared using Bergey’s Manual of Systematic Bacteriology. Initially, fungi were identified based on characteristics such as spores, color, shape, and patterns of colonies on petri plates. Later, lactophenol cotton stain was employed for fungal mounting and identification, following the recommendation of (23). Additionally, for fungi, the isolated fungal aerosols and pure cultures were sent to the Indian Agricultural Research Institute in Pusa, New Delhi, for precise identification. Additionally, fungal spores were recognized based on their shape by Campwell et al. (50) and The British Aerobiological Federation in 1995.

### 2.4 Biochemical characterization of the isolated bacterial samples

Due to the frequent detection of Gram-negative rods in indoor environments, several commercial multi-test kits have been analyzed to recognize members of the family *Enterobacteriaceae* and other harmful microbes. By applying the bacterial colony on a glass slide along with a drop of 3% H_2_O_2_, it was possible to determine if the isolates contained catalase. The appearance of bubbles was considered favorable, however their lack or a few dispersed bubbles were considered bad (24).

#### 2.4.1 IMViC test

IMViC is a set of tests used to identify coliform group microorganisms. IMViC Test Kit (SRL Research Labs Pvt. Ltd.) was used to for the identification of the Gram-negative enteric bacteria which are involved in causing various bacterial diseases. The following test are included in IMViC test.

#### 2.4.2 Indole test

Using Kovac’s and Ehrlich’s reagents, indole production is detected. Red color is produced when indole and the reagent’s aldehyde react (25, 26).

#### 2.4.3 Methyl Red test

The Methyl Red test is used to confirm that enough acid is produced during the fermentation of glucose. A pH indicator called methyl red maintains its red hue at a pH of 4.4 or below. Pyruvic acid is originally created by all enterics through the metabolism of glucose. Some then convert pyruvic acid to other acids, such lactic, acetic, and formic acids, via the mixed acid route. Methyl-red positive bacteria are the ones in question. Later, other enterics metabolize pyruvic acid to neutral end products via the butylene glycol route (25, 26).

#### 2.4.4 Voges Proskauer test

To demonstrate an organism’s capacity to convert pyruvate to acetoin, the Voges Proskauer test is utilized. Butylene glycol is produced via the intermediate acetyl-methyl carbinol (acetoin). Alpha-naphthol and 40% KOH are added to the test broth after incubation and exposure to ambient oxygen. If acetoin is present, it is converted to diacetyl in the presence of air and KOH. In the presence of alphanaphthol, dialdehyde then interacts with the guanidine components of peptone to generate red color. Alpha-naphthol plays the roles of a catalyst and an intensifier of color (25, 26).

#### 2.4.5 Citrate utilization test

This test is designed to assess the microorganism’s capacity to utilize citrate as its only carbon source. Inorganic ammonium salts, which are used as the only source of nitrogen in the media, are present. Oxaloacetate and acetate result from the breakdown of citrate. Pyruvate and CO2 are produced after further breakdown of oxaloactetate. Alkaline pH is the consequence of the production of Na_2_CO_3_ and NH3 from the use of sodium citrate and ammonium salt, respectively. As a result, the medium’s hue changes from green to blue (25, 26).

### 2.5 Molecular analysis of samples (bacterial samples)

#### 2.5.1 DNA extraction

Following the separation and preliminary macroscopic and microscopic identification of the bacteria, each distinctive in shape bacteria was separated into cultures, and the HiPura Bacterial genomic DNA purification Kit (Himedia) was used to extract the DNA from the bacteria.

#### 2.5.2 PCR

In a real-time PCR (Thermo Fischer, USA), the universal bacterial 16S rDNA primers 27F (5′-AGA GTT TGA TCM TGG CTC AG-3′) and 1492R (5′-GGY TAC CTT GTT ACG ACT T-3′) were used to perform polymerase chain reaction (PCR). The reaction was conducted in a 25 μL mixture that contained 1.5 mM MgCl2 (Promega, USA), 0.4 μM of both forward and reverse primer, 0.1 mM of dNTP, 1 × Taq bufer (Thermo Fischer, USA), and one unit of Taq DNA polymerase (Thermo Fischer, USA). Initial denaturation was set at 94°C for 2 min. This was accompanied by 40 cycles of denaturation at 94°C for 1 min, annealing at 50°C for 1 min, elongation at 70°C for 2 min, and final extension at 70°C for 20 min in the thermo-cycle program.

#### 2.5.3 Gel electrophoresis

Gel documentation method was used to visualize amplified DNA following electrophoresis in 1.5% agarose and using ethidium bromide staining. Amplicons were purified with a Himedia gel extraction kit. All the bands apparated on the gel were compared with the standard molecular size of the major airborne bacteria (27).

### 2.6 Meteorological and PM data monitoring

Metrological data was collected from Satyawati College Ashok Vihar Station, 110052, and the Ambient Air Quality Monitoring System of the Delhi Pollution Control Centre (DPCC) interior locations. Relative humidity (RH) (%), wind speed (WS), and temperature (°C) were measured, and their correlation with bioaerosol was ascertained.

### 2.7 Questionnaire survey

A health survey was conducted in the sampling areas using questionnaires with a structured set of questions. A single-stage random sampling procedure was used to choose participants from various locations. The purpose of the questionnaire form was to gather general and health-related data from participants. It was then authorized by Indian Council of Medical Research (ICMR) professionals in a relevant field. To that aim, the form is broken down into four sections. The first section includes demographic information such as name, residence, gender, and age, along with an informed consent form that includes general research details. The volunteer agreement and witness declaration are included in the next section of the form. The form’s next section, the “Indoor Air Quality Questionnaire,” is the most crucial one. It begins with some general information and includes 26 questions on allergies, general health impacts, past allergy medication use, and suggestions for better air quality. A total of 84 households were selected for the health survey and environmental sampling. Household selection followed a single-stage random sampling approach within the defined community clusters. On average, each household had approximately six residents, resulting in a total of 509 individuals who completed the health questionnaire. The response rate was effectively around 100%, as surveys were administered in person by trained research staff. Incomplete or unresponsive entries were excluded to ensure data accuracy. A brief overview of the questionnaire used in the study is provided below:

a) Demographic Information

Age, gender, date of birth, profession, contact details, and duration of residence.

b) Residential Exposure Assessment

- Duration of residence and time spent indoors.
- Subjective assessment of indoor air quality (good, average, poor).
- Proximity to potential pollution sources (industrial areas, water bodies, vacant land).

c) Health History and Symptoms

- Past and present diagnoses of respiratory or allergic conditions (e.g., asthma, bronchitis, allergic rhinitis).
- Frequency and timing of symptoms (e.g., seasonal vs. year-round, time of day).
- Symptom exacerbation due to environmental triggers (dust, mold, weather, pollutants, etc.).

d) Environmental and Lifestyle Factors

- Evidence of moisture/water leakage or renovation activities in the home.
- Lighting adequacy and ventilation quality.
- Occupational exposures or hobbies with potential for air pollutant contact (e.g., welding, auto-repair, farming).

e) Medication and Reproductive History

- Current medication use (e.g., antihistamines, decongestants).
- Childbirth-related outcomes include low birth weight, preterm birth, and neonatal mortality.

f) Workplace Environment Assessment

- Symptoms experienced in the workplace.
- Suspected causes of workplace-related symptoms.
- Observations and suggestions regarding workplace environmental conditions.

### 2.8 Statistical analysis

Statistical analysis and entry of questionnaires was performed using Epi info version 7 and Microsoft Excel. Significance of data (*p-*value) and odds ratio was also analyzed by using the same software. PAST software (v13), Microsoft Excel, and Prizm were used for analyzing the sampling data.

## 3 Results and discussion

### 3.1 Seasonal fluctuations in the overall concentration of bioaerosols

**Figure 3:**
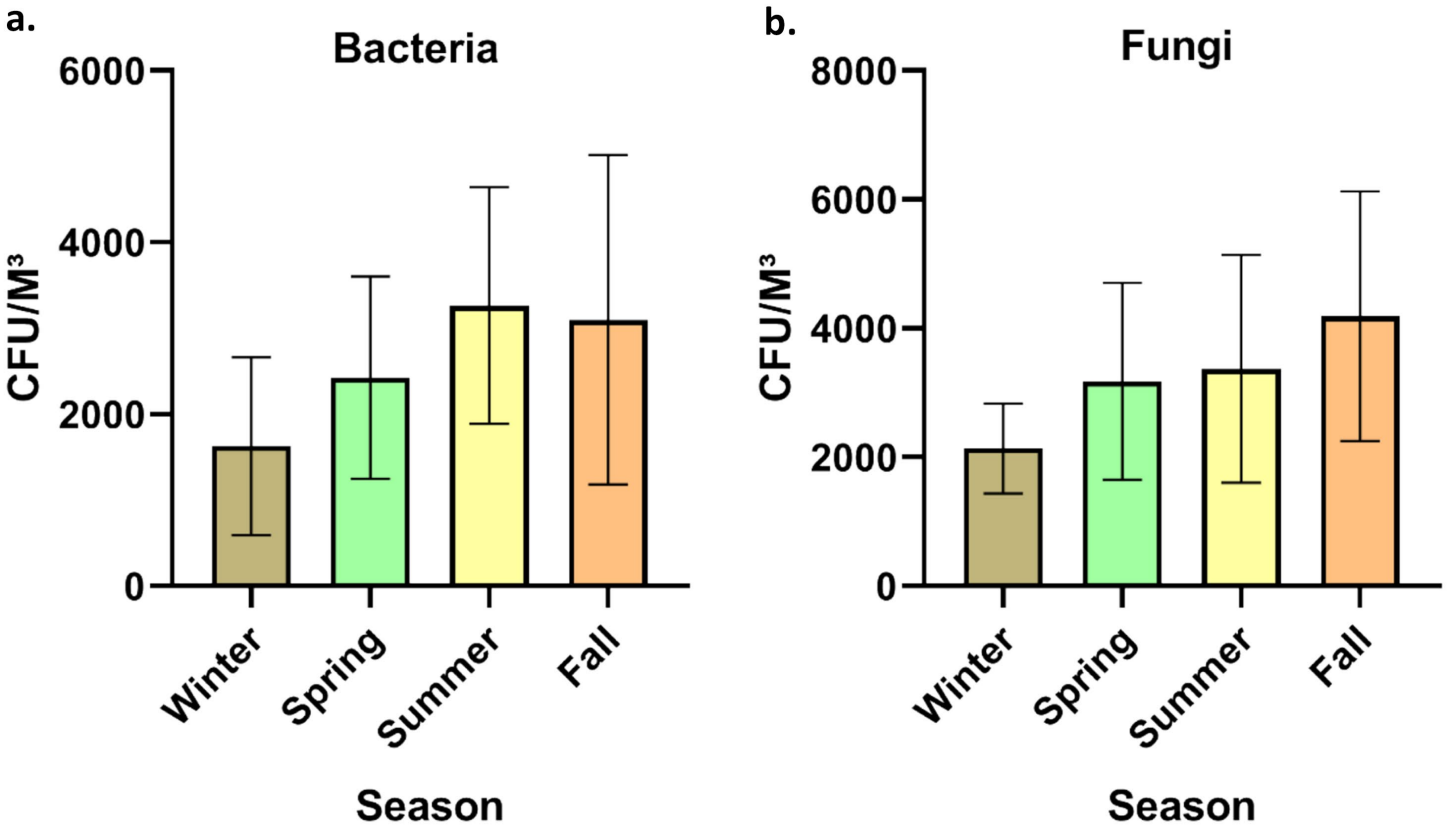
Seasonal concentration of culturable bioaerosols indoor places. **(a)** Bacteria **(b)** Fungi. *n* = 336 samples in all seasons (84 each season); Error bars represent SD of triplicates.

The time allocated for sampling was split into four seasons: winter (December to February), spring (March to May), summer (June to August), and fall (September to November). Concerning bacterial aerosols, the CFU/m^3^ concentration exhibited an upward trend from winter to summer, followed by a subsequent decline in the fall (Figures 3a, 3b). In contrast, fungal aerosol concentration displayed a distinct pattern. The graphical representation indicates a significant rise in fungal spore concentration from winter to spring, with a modest increase from spring to summer. Following this, there was a substantial surge in fungal concentration from summer onwards. It is noteworthy that the total CFU/m^3^ count was greater for fungal aerosols compared to bacterial aerosols. Bacterial aerosol levels varied from 730–5,300 CFU/m^3^, while fungal levels ranged between 1,330–6,050 CFU/m^3^ (Figures 3a, 3b). Pearson correlation matrix between different seasons and microbial concentrations (bacteria and fungi) is provided in Tables 1a,b. The highest levels of bacterial aerosols were observed in August, the final month of summer, as the increased temperature and humidity during this time fostered their growth. Winter had the lowest-ever recorded concentration of bacteria, possibly attributed to lower temperatures and a lack of ventilation. Like bacterial concentrations, it is presumed that fungal CFU levels were highest in the fall due to the favorable combination of temperature and humidity. Consistent with earlier findings from our laboratory, the conditions conducive to the growth of both fungi and bacteria are optimal during the fall season (3) (Table 1).

**Table 1:** Pearson correlation in seasonal microbial concentration in houses (Pearson correlation coefficient) **(a)** Bacteria and **(b)** Fungi.

**(a) Bacteria**	**Winter**	**Spring**	**Summer**	**Fall**
**Winter**	1	0.994	0.860	0.907
**Spring**	0.994	1	0.907	0.860
**Summer**	0.860	0.907	1	0.567
**Fall**	0.907	0.860	0.567	1

**(b) Fungi**	**Winter**	**Spring**	**Summer**	**Fall**
**Winter**	1	0.996	0.832	0.926
**Spring**	0.996	1	0.780	0.956
**Summer**	0.832	0.780	1	0.563
**Fall**	0.926	0.956	0.563	1

Anthropogenic activities, pets, indoor plants and ventilation facilities are among the top factors responsible for the variation in the microbial concentration in indoor areas. Human actions are the primary cause of biological pollution within indoor spaces, as highlighted by Hospodosky et al. (28). According to a study carried out in Delhi, *Cladosporium* was the most prevalent fungus and another fungi were *Aspergillus flavus* and *Ustilago*. The study observed elevated concentrations of fungi from April to July, with reduced concentrations in the cold and arid season (29). Kumari and her colleagues conducted a comparable investigation to assess the fluctuation of fungal aerosols in pig farms across seasons (30). Ascomycota was among the most common phylum of fungi, with Basidiomycota and Zygomycota following closely behind. According to the research, summertime had more variation and quantity than cold (30). A study conducted in multifamily apartments in the USA identified that ventilation, indoor sources, and seasonal variations are the primary factors influencing bioaerosol concentrations within indoor environments (31). According to a recent study in Belgium, insufficient ventilation and filtration systems in indoor spaces could lead to a substantial rise in respiratory pathogens among residents (32). In another study, Karmakar et al. (33) highlighted the prevalence of Ascospore, basidiospore, *Cladosporium,* and *Aspergilli* fungi in elevated concentrations within primary indoor spaces. The availability of nutrients and optimal temperatures favored the proliferation of diverse indoor fungi (33). Seasonal variations have been found in the levels of bacteria and fungi within buildings. Bacterial concentrations were highest in spring at 2165CFU/m^3^ and reached their lowest in summer at 240 CFU/m^3^. Conversely, fungi exhibited their highest concentrations in houses during the summer at 235CFU/m^3^, while their lowest concentrations were recorded in winter at 26 CFU/m^3^ (34). The most recent data, in contrast to this study, shows that the level of bacteria is maximum in August, the start of fall, and least in cold. On the other hand, the trend of fungi prevalence is identical to current study (34). Madhwal et al. (35) measured biological aerosols close to a bus terminal and observed a nearly identical trend across all seasons. As per a study conducted in Dehradun, India, the most common genera of bacteria were *Micrococcus, Staphylococcus,* and *Bacillus,* whereas the most common fungal genera were *Aspergillus, Penicillium,* and *Cladosporium* (35). Airborne fungi exhibited a decline from July to August, possibly attributed to increased soil moisture, leading to reduced fungal resuspension during this time (55, 62). In similar research conducted in China, Summertime was also the season with the greatest populations of bacteria and fungi (36). Recent Chinese research by Li et al. (60) found intriguing variations in indoor microflora between male and female dormitories, revealing higher. Another Indian study reported the bacterial range between 924 and 2,750 CFU/m^3^ however in case of fungi it ranged between 656 and 1799 CFU/m^3^ (37). Considering the current study and other studies, it is interesting to note that most of studies reported the bioaerosol CFU/m^3^ exceeded the recommended limits for the indoor places (38). Hence, there is an urgent need for implementing strict guidelines for improving the microbial indoor air quality.

Supplementary Table S1 illustrates the seasonal variations in different meteorological parameters that may significantly influence bioaerosol composition and concentrations. In addition to presenting meteorological data, the study also statistically correlated them with aerosol concentrations across different seasons (Table 2). Overall, bacterial and fungal aerosol concentrations were found to correlate with these meteorological parameters, with a few exceptions. Previous research from our laboratory, along with other studies, has demonstrated an association between temperature and humidity and bioaerosol concentrations in indoor environments (9, 34).

**Table 2:** Correlation between environmental parameters temperature, relative humidity and wind speed with microbial concentration (a. Temperature T, b. Relative Humidity RH, c. Wind Speed WS).

Parameter		Bacteria		Fungi	
	Season	*p*-value	*R* ^2^	*p*-value	*R* ^2^
T	Winter	0.79	0.62	0.27	0.07
	Spring	0.94	0.88	0.99	0.98
	Summer	−0.99	0.98	−0.84	0.70
	Fall	−0.82	0.67	−1.0	0.99
RH	Winter	−0.75	0.54	−0.2	0.03
	Spring	−0.29	0.08	−0.68	0.45
	Summer	0.97	0.95	0.89	0.80
	Fall	−0.9	0.80	−0.97	0.96
WS	Winter	0.98	0.94	0.94	0.83
	Spring	−0.86	0.90	0.99	0.51
	Summer	−0.38	0.84	−0.57	0.91
	Fall	−0.53	0.47	−0.96	0.98

The fungal aerosol concentrations observed in this study (1330–6,050 CFU/m^3^) significantly exceeded the WHO recommended limit of 500 CFU/m^3^, indicating substantial fungal contamination in indoor environments. Despite the major studies being focused on bacterial aerosols, fungal bioaerosols are equally important and pose considerable health risks, particularly when present in elevated levels. Fine fungal particles (>2.5 μm), which were predominant across seasons, are capable of penetrating deep into the respiratory tract, reaching the bronchioles and alveolar regions. This can trigger allergic responses, exacerbate asthma, and lead to respiratory infections, especially in sensitive populations such as children, the older adult, and immuno-compromised individuals.

The seasonal distribution further highlights the potential health burden. During the rainy season, the dominance of fine fungal particles aligns with increased humidity and favorable conditions for fungal growth and spore dispersal, which may intensify respiratory symptoms like sneezing, wheezing, and allergic rhinitis. Conversely, the aggregation of fungal aerosols with non-biological particles in winter may reduce airborne counts but could still pose risks when resuspended. Although direct correlation with fungal exposure and reported symptoms was not separately analyzed in this study, the consistently elevated fungal levels and particle size distribution strongly suggest a plausible link with the observed respiratory and allergic health complaints among residents.

### 3.2 The pattern of bioaerosol sizes during various seasons

**Figure 4:**
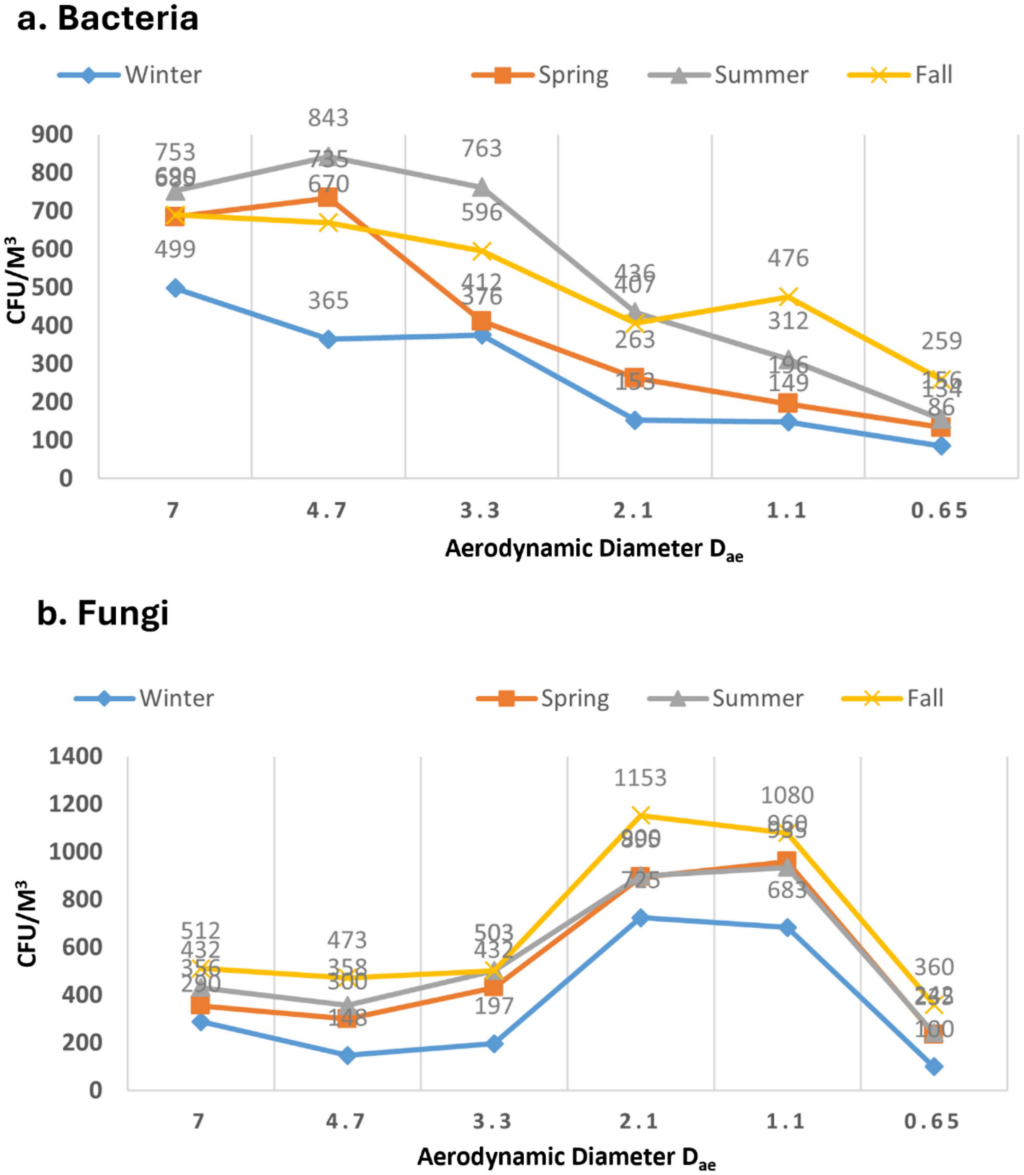
Size distribution in different seasons. **(a)** Bacterial. **(b)** Fungi.

For bacterial samples, in all four seasons, the observed size distribution exhibited a roughly unimodal pattern, peaking at > 4.7 μm during the summer and minimum was recorded in winter peaking at > 3.3 with no statistically significant difference (*p* > 0.05) (Figure 4a). The occurrence of larger particles across various seasons might be attributed to bacterial cell aggregation on non-biological surfaces. In case of all the seasons a rough pattern can be observed which shoes the negative correlation of the CFU with size of the sampler with a few exceptions. Fall was shown to have the greatest level of bacterial aerosols (>0.65 μm) of any seasons (*p* > 0.05). Wintertime dominance of tiny bacterial aerosols implies a shortage of bigger non-biological particles. In a nursery school setting, larger particulate in the (>4.7 μm) range were also discovered by Bragoszewska et al. (39). According to Grzyb and Lenart (40), microorganisms account for more than 70% of all respirable particle fractions. Furthermore, the (>7 μm) fraction had the largest concentrations of both bacteria and fungi particles, according to a Madhwal et al. (35) research from India.

In the context of fungi, there was a distinct trend observed in the size of diameters (*p* > 0.05), as depicted in Figure 4b. The data indicates that particles of smaller fungal sizes (>2.5 μm) were predominant across all seasons. These diminutive particles have the potential to deposit in the lower tracheal or alveolar regions of the lungs, leading to various health issues in humans. The absence of smaller particles in winter could potentially be attributed to the aggregation of fungal aerosols with non-biological particles. The rainy season, however, exhibited a distinctive pattern compared to other seasons. Coarse particles (>7 μm) were minimal, resembling the winter season (*p* > 0.05). On the contrary, fine fungal particles were more prevalent during the rainy season than in any other season. Lal and colleagues documented a similar pattern in the distribution of fungal aerosols, reporting the highest concentrations in the 3rd stage (3.3–4.7 μm) and 4th stage (3.3–2.1 μm) of the Andersen air sampler at various locations in Delhi (41). Previous study published from our laboratory resembled the similar kind of pattern for the distribution of the fungal aerosols in different seasons (3). Recent study from China, reported the similar size distribution pattern results in some of the underground garages in case of the fungal aerosols (36). Typically, microorganisms are linked to the particles found in the air around us in the form of bioaerosols. However, they also have the capability to move independently in the air. Our data indicates that bacterial populations outnumber fungi both in overall concentration and in submicron fractions. Dust formation and aerosolization processes can be triggered by human and anthropogenic activities, impacting the respiratory system in humans (35, 42).

### 3.3 Detection of viable airborne microorganisms

Prior to conducting the molecular analysis of the bacterial samples, extensive biochemical tests were carried out on isolated bacterial genera. Supplementary Tables S2, S3 summarize the biochemical characteristics used to identify general and enteric bacterial genera isolated from samples. Supplementary Table S2 highlights the differentiation of Gram-positive and Gram-negative bacteria based on tests such as catalase, starch hydrolysis, mannitol fermentation, and coagulase activity, helping distinguish genera like *Staphylococcus*, *Streptococcus*, and various bacilli. Supplementary Table S3 focuses on enteric bacteria, using standard IMViC tests (Indole, Methyl Red, Voges-Proskauer, and Citrate) to differentiate species like *Escherichia coli*, *Enterobacter aerogenes*, *Klebsiella pneumoniae*, and *Proteus vulgaris*. These biochemical profiles support accurate genus- and species-level classification of airborne bacterial isolates.

**Figure 5:**
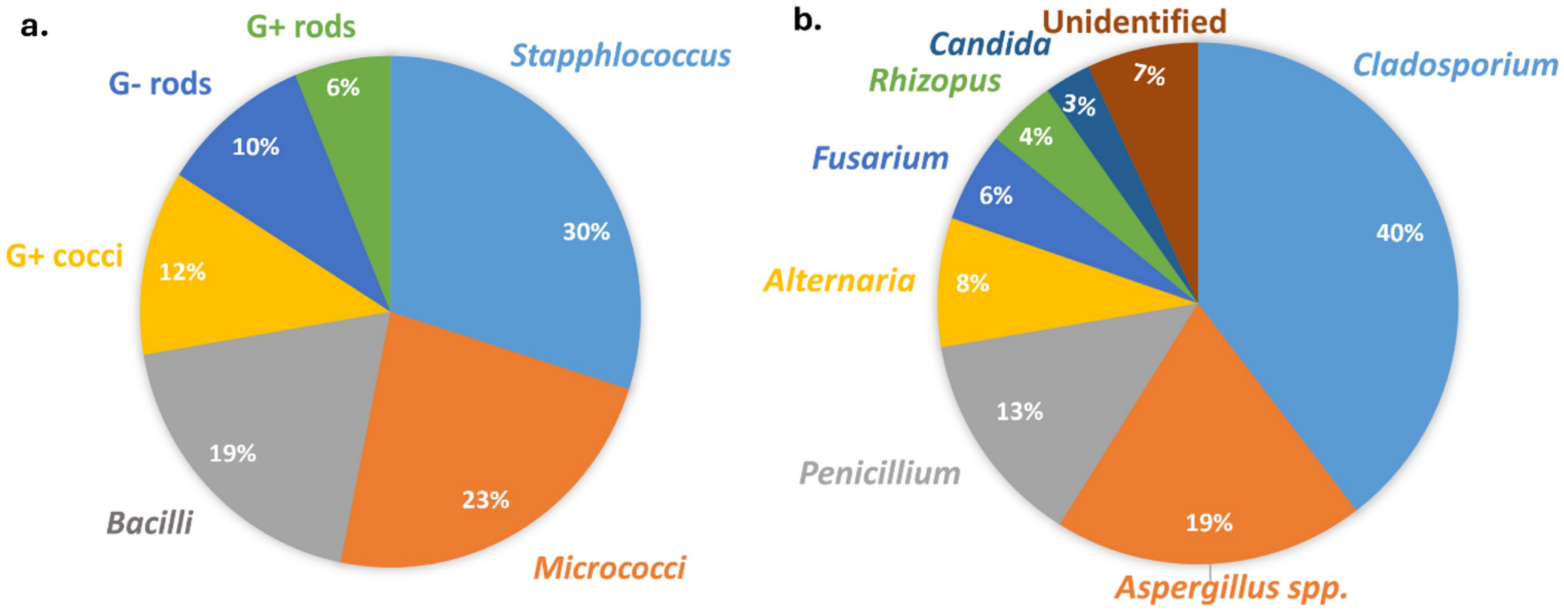
Proportion of microbes isolated from indoor residential houses. **(a)** Bacteria. **(b)** Fungi.

The molecular analysis aimed at identifying bacterial aerosols was conducted in accordance with the procedures outlined in the materials and methods section. Figure 5a presents the major bacterial genera found in all the residences, along with their respective proportions. Notably, the combined concentrations of *Staphylococci*, *Micrococci*, and *Bacilli* accounted for over 70% of the total viable bacterial aerosols.

Fungal aerosol composition in indoor environments was assessed through culture-based identification methods. The identification of conidia from the *Aspergillus* and *Penicillium* groups was verified using culture methods as described by Oliveira et al. (61). Figure 5b illustrates the predominant fungal genera identified in every dwelling, along with their corresponding percentages. Like bacterial aerosols, the three major fungal genera (*Cladosporium*, *Aspergillus* and *Penicillium*) constituted more than 70% of the total culturable fungal aerosols. Like our results, the previous studies also reported the similar composition of fungal aerosols (3, 10, 11). Another recent study from India also identified *Cladosporium* as being present in the highest concentrations in indoor environments (37). Similar to our current research, this study also found nearly the same fungal genera across different houses (37). Various experimental and epidemiological studies also confirmed that these fungal genera isolated from the indoor houses are may also be responsible for the mold allergy (9–11, 43–45). Given the person-to-person transmission of SARS-CoV-2 (COVID-19) through bioaerosols, there is an urgent need for research to quantify the relationship between infected particles and aerosols.

### 3.4 Health impacts resulting from poor microbial indoor air quality identified in questionnaire survey

Participants in the survey were residents of the North-West Delhi sampling locations that were chosen at random. The demographic characteristics of the individuals, including age group, and sex are displayed in Supplementary Table S4. According to the consent agreement, the confidential information of the study participants was not disclosed in the publication. The reason behind selecting these subjects’ houses was to correlate the health effects with the microbial concentration. The study included 509 participants, with a majority being male (67.6%) and the rest female (32.4%). Most subjects were under 30 years old, with 50% below 18 years and 41.3% between 18 and 30 years. As per data, it was notable that more than two-third from the total subjects performed in this study were male. Additionally, around half of the population participated in this study was around 18 years of age.

Bioaerosols can lead to a variety of health problems in humans through inhalation or skin contact. According to the survey, participants experienced the highest incidence of health issues during the winter, while the spring season saw the fewest health problems. Previous studies suggest that the high levels of smoke in Delhi and surrounding areas during winter could be a contributing factor to the increased health effects during this season (3). Interestingly, despite this correlation, the winter months showed the lowest levels of viable bioaerosols in homes.

The symptoms and health effects resulting from exposure to poor indoor air quality have been categorized into respiratory, neural, ocular, bodily pain, and other health issues. These symptoms were identified through an analysis of various questionnaires. Commonly reported problems among participants included frequent coughing, eye irritation, sneezing attacks, headaches, allergies, allergic rhinitis, and chapped lips, as summarized in the Table 3. It was also observed that females exhibited a higher prevalence of these health issues (Table 3). However, conditions such as allergic rhinitis, emphysema, asthma, nasal congestion, wheezing, shortness of breath, sneezing attacks, colds, and hearing problems were more commonly reported in males. According to a 2018 report, females tend to spend more time indoors than males, which could be a likely explanation for their higher susceptibility to health issues linked to indoor air quality (46). Another cross-sectional study highlighted that indoor air pollution in India has a substantial impact on the cognitive functioning of older adult women (47). Notably, many of these prominent symptoms in male participants appear to be occupational health concerns, suggesting that poor air quality in the workplace might be a contributing factor. Interestingly, migraines, headaches, and skin problems were significantly more common in female participants than in males.

**Table 3:** Overall proportion of occurrence of health effects related to indoor air quality in houses.

Symptoms/effects	Gender		
	%M	%F	Total %
Respiratory			
Allergic rhinitis	18.9	16.4	18.1
Emphysema	4.1	0.6	2.9
Asthma	11.6	6.1	9.8
Laryngitis	4.9	3.0	4.3
Bronchitis	3.5	4.8	3.9
Other chest conditions	5.8	12.7	8.1
Sinusitis	5.5	6.7	5.9
Frequent cough	20.1	23.6	21.2
Nasal congestion	11.9	9.7	11.2
Wheezing (except cold)	13.1	9.7	12.0
Sore throat	9.9	12.7	10.8
Shortness of breath	12.5	9.1	11.4
Hoarse voice	5.2	0.6	3.7
Sneezing attacks	20.3	15.2	18.7
Neural			
Migraine	4.7	12.1	7.1
Ocular			
Conjunctivitis	6.7	4.2	5.9
Burning or irritated eyes	21.5	27.3	23.4
Eyes red/watery	14.8	17.0	15.5
Body pain			
Headache	27.3	45.5	33.2
Muscle ache	8.4	11.5	9.4
Neck ache	8.4	14.5	10.4
Other problems			
Multiple colds	7.3	2.4	5.7
Unusual thirst	11.3	17.6	13.4
Chapped lips	23.3	27.9	24.8
Runny nose	6.4	5.5	6.1
Dizziness	6.1	12.1	8.1
Fever (>100.5°F)	7.3	10.3	8.3
Fatigue/Drowsiness	8.4	13.3	10.0
Skin problem	14.0	24.2	17.3
Anxiety	14.0	13.9	13.9
Hearing Problems	14.2	3.6	10.8
Difficulties	10.2	17.0	12.4

Table 4 outlines the overall health issues observed across different groups. In Group A, the most frequently reported symptoms included allergic rhinitis, persistent coughing, sneezing attacks, wheezing, eye irritation, muscle aches, chapped lips, dizziness, and skin and hair problems. Conversely, Group B participants experienced higher rates of asthma, nasal congestion, migraines, and headaches. Interestingly, Groups C and D reported fewer health problems compared to Groups A and B. This difference might be attributed to the fact that younger individuals are more susceptible to health effects and symptoms caused by poor indoor air quality.

**Table 4:** Occurrence of health effects related to indoor air quality in houses in different age groups.

Symptoms/effects	Age in years				Total
	0–18	18–30	30–45	45+	
Respiratory					
Allergic rhinitis	21.2	15.7	12.5	8.3	18.1
Emphysema	2.0	3.8	6.3	8.3	3.1
Asthma	10.2	10.5	3.1	8.3	9.8
Laryngitis	3.9	4.8	3.1	8.3	4.3
Bronchitis	2.7	5.7	3.1	0.0	3.9
Chest congestion	6.7	6.2	12.5	8.3	6.9
Sinusitis	6.3	6.2	0.0	0.0	5.7
Frequent cough	23.1	20.0	15.6	16.7	21.2
Nasal congestion	10.2	14.3	0.0	8.3	11.2
Wheezing	13.7	11.0	3.1	0.0	11.6
Sore throat	12.9	8.6	0.0	16.7	10.4
Shortness of breath	12.9	10.5	0.0	8.3	11.0
Hoarse voice	5.1	2.9	0.0	16.7	4.1
Sneezing attacks	15.7	13.8	9.4	8.3	14.3
Neural					
Migraines	8.2	10.5	6.3	8.3	9.0
Ocular					
Conjunctivitis	5.9	6.2	6.3	0.0	5.9
Burning or irritated eyes	31.4	20.0	0.0	8.3	24.2
Eyes red/watery	18.0	13.3	3.1	8.3	14.9
Body aches					
Headache	32.5	34.8	12.5	33.3	32.2
Muscle ache	13.3	9.5	0.0	8.3	10.8
Neck ache	12.2	8.6	0.0	0.0	9.6
Other problems					
Multiple colds	8.6	7.6	3.1	0.0	7.7
Unusual thirst	13.3	14.3	6.3	16.7	13.4
Chapped lips	28.2	23.8	6.3	16.7	24.8
Runny nose	7.1	6.2	0.0	8.3	6.3
Dizziness	11.4	6.7	0.0	0.0	8.4
Fever (>100.5°F)	10.6	8.6	0.0	0.0	8.8
Fatigue/Drowsiness	11.0	10.0	6.3	0.0	10.0
Skin problem	20.0	18.1	3.1	0.0	17.7
Anxiety	15.7	11.0	0.0	8.3	12.6
Hearing problems	7.8	2.9	0.0	0.0	5.1
Difficulties in breathing	15.7	11.9	0.0	0.0	12.8

According to the overall seasonal data, the majority of people experience one or more health issues during the fall, while the fewest are affected in the spring. For the assessment of seasonal health issues related to poor indoor air quality, subjects were classified into six distinct categories (Table 5). In addition to those experiencing health problems during specific seasons, two additional categories were established: one for individuals suffering from health issues year-round, and another for those experiencing health problems in more than one season. Health effects caused due to poor microbial indoor air quality are significantly correlated with the seasons. In addition to inquiring about health-related issues, a question regarding the quality of indoor air was also asked (Supplementary Figure S1). According to a study, people are less likely to report the outdoor air pollution related health issues than indoor air pollution. However, it is well known that the indoor air pollution is more crucial as most of the time spent by the people is indoor areas (48).

**Table 5:** Occurrence of health effects related to indoor air quality in houses in different seasons.

Symptoms/effects	Season					
	Winter	Spring	Summer	Fall	Year round	> One season
Respiratory						
Allergic rhinitis	17.9	15.3	16.2	19.6	25.0	25.0
Emphysema	10.3	2.9	3.7	2.9	6.3	0.0
Asthma	15.4	12.1	6.5	4.4	4.2	15.6
Laryngitis	7.7	2.3	2.8	7.4	8.3	9.4
Bronchitis	7.7	0.5	6.5	2.9	4.2	12.5
Other chest conditions	10.3	1.9	7.5	8.8	14.6	18.8
Sinusitis	15.4	1.4	3.7	7.4	8.3	12.5
Frequent cough	17.9	15.3	17.8	26.5	29.2	31.3
Nasal congestion	12.8	9.8	7.5	13.2	12.5	18.8
Wheezing (except cold)	9.8	5.1	11.2	14.7	12.5	18.8
Sore throat	15.4	8.8	12.1	8.8	6.3	12.5
Shortness of breath	10.3	8.4	8.4	17.6	12.5	15.6
Hoarse voice	4.7	2.6	1.9	5.9	2.1	3.1
Sneezing	20.5	11.6	15.0	20.6	6.3	34.4
Neural						
Migraines	10.3	5.1	5.6	10.3	4.2	15.6
Ocular						
Conjunctivitis	10.3	3.7	5.6	5.9	12.5	3.1
Burning or irritated eyes	21.9	10.3	24.3	32.4	20.8	28.1
Eyes red/watery	14.0	10.3	12.1	25.0	18.8	25.0
Body aches						
Headaches	25.6	14.0	25.2	20.6	25.0	31.3
Muscle ache	23.1	11.6	7.5	10.3	6.3	9.4
Neck ache	10.7	2.6	5.6	13.2	10.4	6.3
Other problems						
Multiple colds	6.5	5.1	5.6	10.3	6.3	18.8
Unusual thirst	5.1	5.1	21.5	19.1	12.5	43.8
Chapped lips	25.6	17.2	26.2	35.3	25.0	28.1
Dizziness	9.8	5.1	9.3	4.4	10.4	6.3
Fever (>100.5°F)	12.8	6.5	8.4	13.2	10.4	6.3
Fatigue/Drowsiness	7.9	5.1	16.8	13.2	6.3	6.3
Wheezing	4.2	2.6	3.7	1.5	8.3	6.3
Skin problem	23.1	17.2	16.8	20.6	14.6	28.1
Anxiety	12.1	12.8	14.0	16.2	12.5	31.3
Hearing problems	7.4	5.1	2.8	4.4	6.3	3.1
Difficulties	17.9	10.2	15.0	19.1	10.4	3.1

The predominance of fine fungal aerosols, particularly in stages 4–5 (particle sizes >2.5 μm), suggests a high potential for deep lung penetration, notably into the tracheobronchial and alveolar regions (3, 9). These regions are more susceptible to chronic inflammatory responses due to the deposition of respirable particles. Several reported health complaints, such as persistent coughing, shortness of breath, wheezing, and chest discomfort, are consistent with lower respiratory tract irritation, which aligns with exposure to fine fungal particles. Inhalation of such particles can trigger or exacerbate conditions like asthma, hypersensitivity pneumonitis, and allergic bronchopulmonary mycosis, particularly in sensitized individuals or those with pre-existing respiratory conditions. Moreover, the higher abundance of fine particles during the rainy season could correlate with increased fungal sporulation and dispersal due to elevated humidity levels. The aggregation of fungal aerosols with non-biological particles during winter, resulting in fewer respirable-sized particles, may explain the relative reduction in lower respiratory symptoms during that season (56). These results highlight the critical role of particle size in evaluating health risks linked to bioaerosol exposure and emphasize the need for improved health questionnaires in future studies to more accurately capture symptoms associated with specific regions of the respiratory tract.

Although bioaerosol concentrations were found to be lowest during the winter season (Figure 3), the highest prevalence of respiratory and general health symptoms was reported during this period (Table 5). This apparent contradiction can be explained by several confounding factors. During winter, residents tend to remain indoors for extended periods with limited ventilation, leading to prolonged exposure to indoor pollutants. Additionally, increased indoor crowding in small, poorly ventilated homes can enhance the transmission of airborne pathogens. Moreover, winter in Delhi is associated with severe outdoor air pollution events, including smog episodes driven by high levels of particulate matter (PM2.5), nitrogen oxides, and emissions from biomass burning and vehicular traffic. These pollutants can penetrate indoor environments and exacerbate respiratory symptoms. Therefore, the heightened health effects observed in winter likely result from a combined influence of indoor crowding, poor ventilation, and elevated levels of non-biological pollutants rather than bioaerosols alone.

### 3.5 Policy recommendations to improve indoor air quality and public health

To improve indoor air quality (IAQ) and safeguard public health, a comprehensive set of strategies should be implemented across various indoor environments (51, 52). One of the most critical recommendations is the enhancement of ventilation and air filtration systems. The integration of high-efficiency particulate air (HEPA) filters into ventilation systems or the use of standalone filtration units can substantially reduce airborne particulate matter and microbial loads, including bacteria, fungi, and viruses (49). Another key intervention involves the use of low-emission construction and furnishing materials, which are known to release fewer volatile organic compounds (VOCs) and support lower microbial growth rates. Such materials contribute to a more stable and less contaminated indoor environment, particularly in high-density or poorly ventilated settings (50). For community housing and commercial buildings, it is essential to adopt mandatory indoor air quality (IAQ) standards, such as ASHRAE 62.1. Low-cost air purifiers offer an affordable and practical solution for improving indoor air quality, especially in resource-limited settings. They can effectively reduce particulate matter and airborne microbes in polluted and poorly ventilated environments. Additionally, maintaining appropriate air exchange rates in high-occupancy areas, such as gyms, lobbies, and meeting spaces, is crucial for minimizing the risk of airborne disease transmission within indoor environments.

Furthermore, it is essential to support and expand ongoing scientific research aimed at establishing evidence-based bioaerosol exposure thresholds and region-specific IAQ guidelines. These efforts should include comprehensive monitoring of microbial contaminants across different building types and climate zones to inform public health policies and building design standards. Implementing these measures in an integrated manner will significantly improve indoor air quality, lower the risk of respiratory diseases, and contribute to better overall health and well-being of building occupants.

## 4 Conclusion

The current study demonstrated that indoor environments in Delhi exhibit higher microbial concentrations than outdoor settings, particularly during the summer and fall seasons. The prevalence of common health symptoms such as headaches and chronic allergies was positively associated with elevated microbial levels and influenced by meteorological factors like temperature and humidity. Human activity and ventilation patterns were also found to significantly shape the diversity and abundance of airborne biological particles indoors.

However, this study has several limitations. The data were collected from selected urban locations in Delhi, which may not represent broader geographic or climatic variations. Therefore, generalizing the findings to other regions requires caution, as seasonal and climatic factors affecting indoor air quality may vary significantly across different global contexts. Moreover, while this study established associations between bioaerosol concentrations and self-reported health symptoms, direct causal links could not be confirmed. There is also a need for methodological refinement, particularly in exposure assessment approaches across diverse indoor environments and population groups. The reliance on culture-based methods may have underestimated the total microbial diversity. Furthermore, future studies should account for the time participants spend indoors to better assess household bioaerosol exposure and establish accurate dose–response relationships. Given that symptoms observed in this study may also be influenced by general ambient air quality, comprehensive exposure assessment, including both indoor and outdoor environments, is essential.

Future studies should include longitudinal exposure assessments, incorporate culture-independent techniques such as metagenomics, and involve diverse geographic regions to better understand global patterns. Additionally, interdisciplinary efforts integrating microbiology, environmental science, and public health are essential for developing evidence-based indoor air quality guidelines.

## Data Availability

The original contributions presented in the study are included in the article/Supplementary material, further inquiries can be directed to the corresponding author.
