# Supplementary material for: Microbial indoor air pollution in Delhi Metropolitan City is attributable to severe respiratory and general health effects among residents
Source: Front Public Health. 2025 Jul 24;13:1626827. doi: 10.3389/fpubh.2025.1626827 (PMC12328385; doi:10.3389/fpubh.2025.1626827)
Supplement: Supplementary file 1 [file Table_1.docx]

Supplementary material to

**Microbial Indoor Air Pollution in Delhi Metropolitan City is Attributable to Severe Respiratory and General Health Effects among Residents**

Pradeep Kumar^1,3^, Rajeev Singh^1,2^*

1. Department of Environmental Studies, Satyawati College, University of Delhi, Delhi-52, India

2. Department of Environmental Science, Jamia Millia Islamia, New Delhi. 110025, India

3. Department of Agricultural and Biosystems Engineering, South Dakota State University, Brookings, SD 57007, USA

**Email-** [10rsingh@gmail.com](mailto:10rsingh@gmail.com)

**Correspondence address-** Dr. Rajeev Singh, Department of Environmental Science, Jamia Millia Islamia, Jamia Nagar, New Delhi 110025, India

**Orcid ID-**

**Pradeep Kumar- 0000-0001-7245-8211**

**Rajeev Singh- 0000-0001-8804-155X**

**Table S1-** Seasonal variation in meteorological parameters in houses (a. Temperature T, b. Relative Humidity RH, c. Wind Speed WS)

| Season | Month | T | RH | WS |
| --- | --- | --- | --- | --- |
| Winter | Dec | 14.4 | 75.4 | 2.7 |
|  | Jan | 12.8 | 83.6 | 4.7 |
|  | Feb | 17.6 | 62.8 | 6.5 |
| Spring | Mar | 25.3 | 55.8 | 5.7 |
|  | Apr | 31.1 | 35.8 | 5.5 |
|  | May | 33.4 | 48.2 | 4.1 |
| Summer | Jun | 34.6 | 46.6 | 9.2 |
|  | Jul | 30.8 | 78.3 | 6.1 |
|  | Aug | 30.5 | 76.7 | 6.8 |
| Fall | Sep | 29.4 | 76.9 | 5.5 |
|  | Oct | 24.9 | 74.2 | 3.6 |
|  | Nov | 20 | 69.1 | 2.6 |

**Table S2.** Biochemical characterization of general bacterial genera

| **Biochemical Tests** | **Suspected Bacterial Species** | | | |
| --- | --- | --- | --- | --- |
|  | **G+Bacilli** | **G+Cocci** | **G-Cocci** | **G-Bacilli** |
|  |  | ***Staphylococcus*** | ***Streptococcus*** |  |
| **Catalase** | **+** | **+** | **-** | **+/-** |
| **Starch Hydrolysis** | **+** | **-** | **+** | **-** |
| **Acid Production from Mannitol** | **+** | **+** | **-** | **+** |
| **Nitrate reduction** | **+** |  |  |  |
| **H_2_S** | **+** | **-** | **-** | **+/-** |
| **Urease** |  | **+** |  |  |
| **Oxidase** |  | **-** | **+/-** |  |
| **Coagulase** | **-** | **+** |  |  |
| **Lipage** |  | **+** |  |  |

**Table S3.** Biochemical characterization of the Enteric bacteria

| **Biochemical Test** | **Bacterial species** | | | |
| --- | --- | --- | --- | --- |
|  | ***Escherichia coli*** | ***Enterobacter aerogenes*** | ***Klebsiella pneumoniae*** | ***Proteus vulgaris*** |
| **Indole Test** | **+** | **-** | **-** | **+** |
| **Methyl red Test** | **+** | **-** | **+/-** | **+** |
| **Voges Proskauer Test** | **-** | **+** | **+** | **-** |
| **Citrate Utilization** | **-** | **+** | **+** | **+/-** |

**Table S4**: Demographic profile of the subjects involved in the study.

| Demographical features Number of Subjects % of Subjects  *Gender*  Male 344 67.6  Female 165 32.4  *Age groups*  < 18 Years 255 50.0   - 1. Years 210 41.3   2. Years 32 6.3   > 45 Years 12 2.4 |
| --- |

**Fig S1.** Feelings of People regarding satisfaction related to indoor air quality
